# Semaphorin 3A is a marker for disease activity and a potential immunoregulator in systemic lupus erythematosus

**DOI:** 10.1186/ar3881

**Published:** 2012-06-14

**Authors:** Zahava Vadasz, Tharwat Haj, Katalin Halasz, Itzhak Rosner, Gleb Slobodin, Dina Attias, Aharon Kessel, Ofra Kessler, Gera Neufeld, Elias Toubi

**Affiliations:** 1 Division of Allergy and Clinical Immunology, Bnai-Zion Medical Center, Rappaport Faculty of Medicine. Technion, Haifa-31048, Israel; 2 Division of Rheumatology, Bnai-Zion Medical Center, Rappaport Faculty of Medicine. Technion, Haifa-31048, Israel; 3 Division of Haematology, Bnai-Zion Medical Center, Rappaport Faculty of Medicine. Technion, Haifa-31048, Israel; 4 The Cancer and Vascular Research Biology Center, Rappaport Faculty of Medicine. Technion, Haifa-31096, Israel

## Abstract

**Introduction:**

Semaphorin 3A (sema3A) and neuropilin-1 (NP-1) play a regulatory role in immune responses and have a demonstrated effect on the course of collagen induced arthritis. This study was undertaken to evaluate the role of sema3A and NP-1 in the pathogenesis of systemic lupus erythematosus (SLE) and the specific effect of sema3A on the auto-reactive properties of B cells in SLE patients.

**Methods:**

Thirty two SLE and 24 rheumatoid arthritis (RA) patients were assessed and compared with 40 normal individuals. Sema3A serum levels were measured and correlated with SLE disease activity. The *in vitro *effect of sema3A in reducing Toll-like receptor 9 (TLR-9) expression in B cells of SLE patients was evaluated.

**Results:**

Sema3A serum levels in SLE patients were found to be significantly lower than in RA patients (55.04 ± 16.30 ng/ml versus 65.54 ± 14.82 ng/ml, *P *= 0.018) and lower yet than in normal individuals (55.04 ± 16.30 ng/ml versus 74.41 ± 17.60 ng/ml, *P *< 0.0001). Altered serum sema3A levels were found to be in inverse correlation with SLE disease activity, mainly with renal damage. The expression of both sema3A and NP-1 on B cells from SLE patients was significantly different in comparison with normal healthy individuals. Finally, when sema3A was co-cultured with cytosine-phosphodiester-guanine oligodeoxynucleotides (CpG-ODN)-stimulated B cells of SLE patients, their TLR-9 expression was significantly reduced, by almost 50% (*P *= 0.001).

**Conclusions:**

This is the first study in which a reduced serum level of sema3A was found in association with SLE disease activity. It also raises the possibility that sema3A may have a regulatory function in SLE.

## Introduction

Semaphorins are a large family (classified into eight subclasses) of secreted and membrane bound proteins originally discovered in the nervous system, which are involved in repulsive axon guidance during nervous system development [1-3]. They relate to two families of receptors: the neuropilins (NP-1 and NP-2) as the primary ligand binding sites and plexins, as the signal-transducing components [4-6]. Recent data have pointed to the involvement of NP-1 and semaphorins in the regulation of the immune system, and are thus denoted as 'immune semaphorins' [7-9].

Semaphorin 3A (sema3A), a secreted member of this family, is well reported as a potent immuno-regulator during all immune response stages, namely, the early initiation and the late phase of inflammatory processes [10]. The expression of sema3A, NP-1, NP-2, and plexins were found to be increased on differentiating macrophages and on activated T cells, suggesting that they have a role in modulating inflammatory conditions [11]. The addition of sema3A to dendritic cell (DC)/T cell co-cultures significantly inhibited allogeneic T cell proliferation. Moreover, neutralization by blocking antibodies against endogenous sema3A resulted in a profound increase in T cell proliferation [12]. Both NP-1 and sema3A expression was recognized on T regulatory cells as a suppressive marker, contributing to the regulatory properties of these cells [13-16]. In another study, the known immunosuppressive function of bone marrow mesenchymal stem cells (MSCs) was attributed in part to their ability to secrete sema3A which, by binding to NP-1, was found to inhibit T cell proliferation [17]. Becoming a frontier player in the regulation of immune responses and the maintenance of self-tolerance, sema3A should be expected to be involved in the pathogenesis of many autoimmune diseases [18,19]. The study by Catalano [20] was the first to report on the defective expression of sema3A in CD4 T cells derived from patients with rheumatoid arthritis (RA). In this study, sema3A was shown to enhance the suppressive ability of CD4^+^NP-1^+ ^T cells by increasing their IL-10 expression and their regulatory function on effector CD4^+ ^T cells. Their altered expression on T cells was shown to correlate with the progression of RA. In consideration of the above, we initiated this current study to assess the possible role of sema3A in systemic lupus erythematosus (SLE), namely to measure its serum level in SLE patients and assess whether this level correlates with disease status. In addition, we sought to assess the possible *in vitro *immuno-modulatory effect of sema3A on B cells of SLE patients.

## Materials and methods

### Patient population

Serum samples from 32 SLE patients and from 24 RA patients (disease control) who were followed in the out-patient clinics of an academic community hospital, and from 40 healthy controls, were all analyzed for sema3A serum level. SLE patients were evaluated for disease activity by using the Systemic Lupus Erythematosus Disease Activity Index (SLEDAI) [21]. All SLE patients were scored mild to severe with a SLEDAI ranging between 4 and 24. Of relevance, blood was drawn before any steroid pulse or increase of cytotoxic therapy was initiated when severity of disease activity required it. Informed consent was obtained, and the study was approved by the local Helsinki committee. Table 1 summarizes the clinical and laboratory characteristics of all SLE patients included in this study. Seven patients with mild disease were treated only with hydroxychloroquine (HCQ), another eight patients were treated with add-on 2.5 to 5 mg of prednisone, and 17 patients, considered having moderate to severe disease, received low to moderate daily doses of cytotoxic therapy. Nine patients suffered from renal involvement, 19 patients were anti-ds DNA antibody positive and 17 had anti-cardiolipin antibodies of moderate to high titers.

**Table 1:** Clinical characteristics of SLE patients

	SLEDAI	Sema3A (ng/ml)	a. Cal	LN	a. dsDNA	Complement		Treatment
							
						C3	C4	
1	16	44		**+**	**+**	L	L	HCQ; P
2	14	32	**+**	**+**	**+**	L	L	HCQ; P
3	3	82				N	N	HCQ
4	4	63				N	L	P
5	4	69	**+**			N	N	HCQ; P; AZA
6	4	51	**+**			N	N	HCQ; Atabrin
7	5	69			**+**	N	L	HCQ; P
8	8	53				N	L	HCQ; P; AZA
9	6	79	**+**		**+**	L	L	HCQ; P; AZA
10	12	44				N	L	HCQ; P; AZA
11	6	65				N	N	HCQ; P
12	9	48			**+**	N	L	HCQ; P; AZA
13	6	65	**+**		**+**	L	L	HCQ; P; AZA
14	3	76				N	L	HCQ; P
15	12	42	**+**		**+**	N	L	HCQ; P; AZA
16	8	64	**+**		**+**	L	L	HCQ; P;
17	18	38	**+**	**+**	**+**	L	L	HCQ; P; AZA
18	18	33	**+**	**+**	**+**	L	L	HCQ; P; MTX; IVIg
19	4	68			**+**	N	L	HCQ; P; AZA
20	6	55				N	N	HCQ; P
21	6	60	**+**		**+**	N	L	HCQ; P
22	24	20	**+**	**+**	**+**	L	L	HCQ; P; MTX; IVIg
23	5	70	**+**			N	N	HCQ; P
24	10	50	**+**	**+**	**+**	L	L	HCQ; P; AZA
25	13	36	**+**	**+**	**+**	L	L	HCQ; P;
26	4	65			**+**	N	N	HCQ;
27	26	26	**+**	**+**	**+**	L	L	HCQ; P; CYC
28	6	61				N	N	HCQ; P; AZA
29	4	63				N	N	HCQ; P; AZA
30	4	59				N	L	HCQ
31	5	58	**+**		**+**	N	N	HCQ; P; AZA
32	20	34	**+**	**+**	**+**	L	L	HCQ; P; CYC

a.Cal, anti-cardiolipin; a.dsDNA, anti-double-stranded DNA; AZA, azathioprine; CYC, cyclophosphamide; F, female; HCQ, hydroxychloroquine; IVIg, intravenous immunoglobulin; L, low; LN, lupus nephritis; M, male; MTX, Methotrexate; N, normal; P, prednisone; Sema3A, semaphorin 3A.

### Semaphorin 3A serum level

The measurement of sema3A serum level was conducted using a commercial ELISA kit (USCNK Life Science, Wuhan, P.R.China) according to the manufacturer's instructions. The serum samples were stored at -20° until ELISA evaluation.

### Clinical correlations

Results of the SLE patients' serum sema3A levels were correlated with their SLEDAI score, renal involvement and laboratory serologic studies including anti-dsDNA, anti-cardiolipin antibodies and C3-C4 serum levels.

### The expression of Semaphorin 3A and NP-1 on CD25^high ^B cells

The expression of sema3A on CD19/CD25^high ^B cells from healthy controls and SLE patients was assessed by staining whole blood with monoclonal antibodies, human anti-CD19 FITC/PE and CD25 PC5 (Immunotech, Beckman-Coulter, Marseille, France), and human anti-sema3A AlexaFluor 488 or human anti-NP1 PE (R&D, Minneapolis, MN, USA), and evaluated using flow cytometry software (FC500 and CXP software, Beckman Coulter, Brea, CA, USA).

### Purification of B cells

B cells were purified from peripheral blood of SLE patients. To do so, peripheral blood mononuclear cells (PBMCs) were isolated on Lymphoprep (Axis-Shield, Oslo, Norway), and B lymphocytes were isolated by positive selection using CD22 microbeads (20 μl/10^7 ^cells; Miltenyi Biotec, Bergisch Gladbach, Germany) according to the manufacturer's instructions, achieving > 98% purity.

### Toll-Like-Receptor 9 (TLR-9) expressions

Purified B cells, gating on memory CD19+/CD27+ (Immunotech, Beckman-Coulter, Marsellie, France) B cells, from SLE patients were analyzed for TLR-9 expression. Condition-media from HEK293- cells, which were infected by NSPI-CMV-FLAG lentivirus with or without sema3A cDNA, as previously described [22], was added to purified B cells activated by, cytosine-phosphodiester-guanine oligodeoxynucleotides (ODN-CpG) and incubated for 60 hours. After incubation, cells were fixed and permeabilized using a commercial kit ('Fix and Perm', Invitrogen, Carlsbad, CA, USA), according to the manufacturer's instructions, and analyzed for TLR-9 expression. The staining was performed using specific monoclonal anti-human TLR-9-PE antibody (Imgenex, San Diego, CA, USA) and evaluated using a FC500 flow cytometer.

### Statistical analysis

Comparison of sema3A expression on B cells of SLE patients and healthy controls was performed using the unpaired student t-test. The correlation coefficient (r) of clinical correlation between SLEDAI score and sema3A levels was determined using the Pearson correlation test. A two tailed *P*-value of 0.05 or less was considered to be statistically significant.

## Results

### Semaphorin 3A serum level

**Figure 1:**
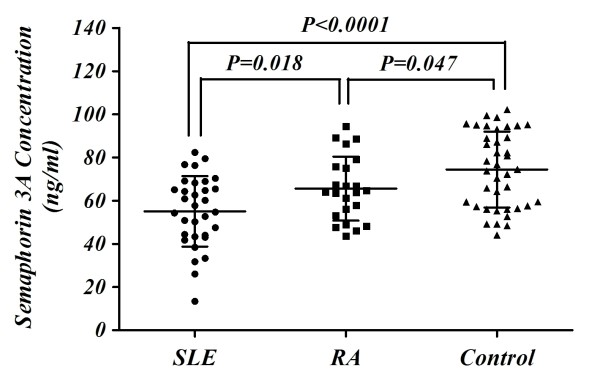
**Serum levels of semaphorin 3A in SLE, RA patients and healthy controls**. Sema3A serum levels were significantly lower in SLE patients compared to those in RA patients (55.04 ± 16.30 ng/ml versus 65.54 ± 14.82 ng/ml, *P *= 0.018) and even lower compared to healthy controls (55.04 ± 16.30 ng/ml versus 74.41 ± 17.60 ng/ml, *P *< 0.0001). RA, rheumatoid arthritis; SLE, systemic lupus erythmatosus.

As shown in Figure 1, the sema3A serum level was lower in RA and SLE patients compared to healthy controls. In controls, the serum level was 74.41 ± 17.60 ng/ml whereas in SLE patients it was significantly lower (55.04 ± 16.30 ng/ml, *P *< 0.0001). The sema3A level was also low in RA patients (65.54 ± 14.82 ng/ml, *P *= 0.047) compared to its level in healthy controls but was higher than that of SLE patients (*P *= 0.018).

### Clinical correlations

**Figure 2:**
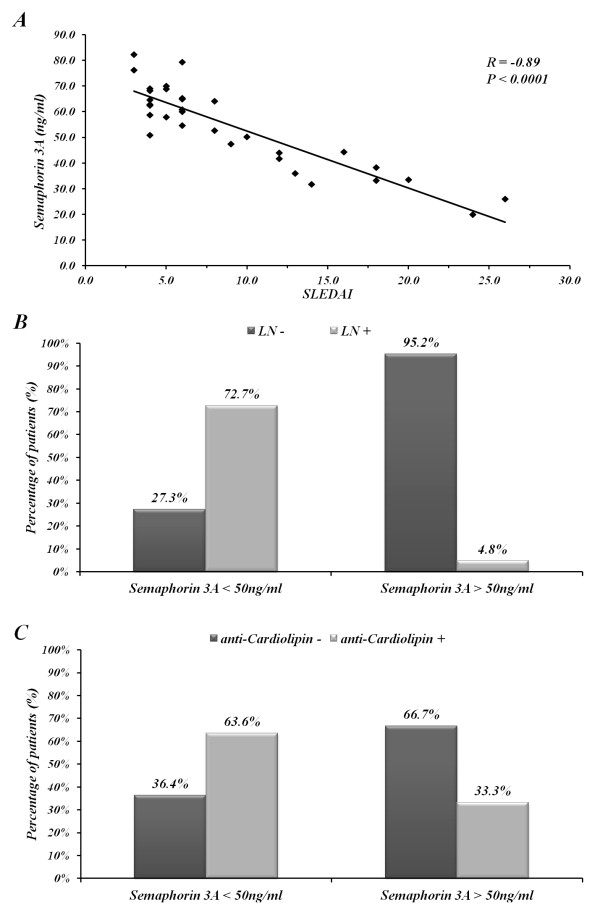
**Clinical correlations**. (**A**) demonstrates the inverse correlation between sema3A serum levels in SLE patients and their SLEDAI score (R = **-**0.89, *P *< 0.0001). (**B**) expresses the association between sema3A serum level and kidney involvement. As is shown, serum levels higher than 50 ng/ml are associated with a lower rate of kidney involvement, and those with higher than 50 ng/ml are associated with increased rate of renal damage. (**C**) shows the association between decreased serum sema3A levels and the presence of anti-cardiolipin antibodies positivity. Sema3A, semaphorin 3A; SLEDAI, Systemic Lupus Erythematosus Disease Activity Index.

**Figure 3:**
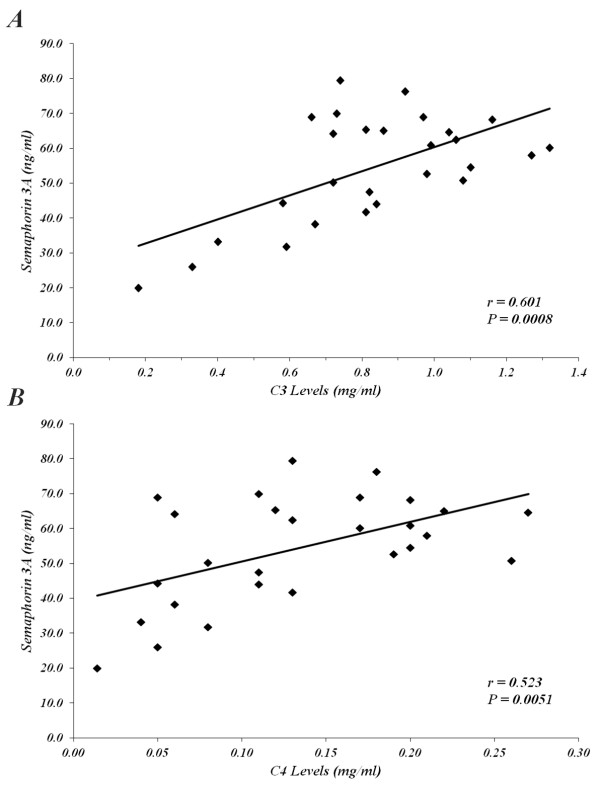
**The correlation of C3-C4 levels with sema3A serum levels**. **(A**) demonstrates a positive correlation between sema3A serum levels in SLE patients and C3 values (r = 0.078, *P *= 0.0008). **(B**) demonstrates a positive correlation between sema3A serum levels in SLE patients and C4 values (r = 0.523, *P *= 0.005). Sema3A, semaphorin 3A; SLE, systemic lupus erythematosus.

Sema3A serum levels in SLE patients were evaluated in relation to SLE disease activity determined by SLEDAI score (including anti-dsDNA antibody). The levels were found to be inversely correlated with disease activity (R = **-**0.89, *P *< 0.0001) (Figure 2A). In addition, the association between the sema3A serum levels and the presence of lupus nephritis was evaluated: 73% of patients with a sema3A level below 50 ng/ml had kidney involvement. However, in those with sema3A levels above 50 ng/ml, only 5% had kidney involvement (Figure 2B). Further, lower sema3A serum levels were also found in association with the higher presence of anti-cardiolipin antibodies; namely, when the sema3A level measured was below 50 ng/ml, 64% of the patients were anti-cardiolipin positive; however, with sema3A levels above 50 ng/ml, only 33% of patients were anti-cardiolipin positive (Figure 2C). Anti-beta 2 Glycoprotein1 (GPI) was performed in all positive anti-cardiolipin positive patients, and was found positive in only ten patients (a too small number for a proper correlation). However, when we looked for a possible correlation between sema3A and the presence of thrombotic events we found that among all anti-cardiolipin positive patients, five developed thrombotic events (patients No. 1, 15, 22, 25, 32), all of whom had very low sema3A levels, and in three of whom anti-beta2 GPI was also positive. In addition, a positive correlation was found between the serum level of sema3A and C3 values (r = 0.078, *P *= 0.0008) and C4 values (r = 0.523, *P *= 0.005) (Figure 3A and 3B).

### The expression of Semaphorin 3A and NP-1 on B cells

**Figure 4:**
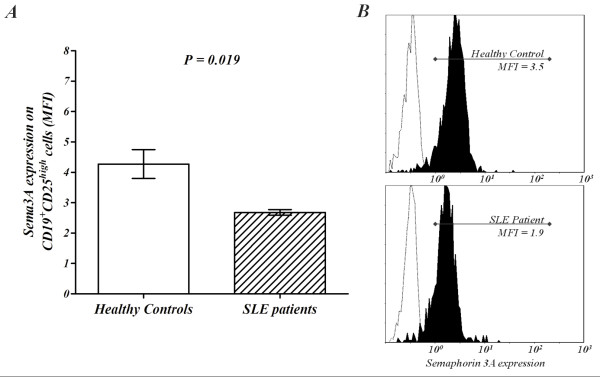
**Expression of Semaphorin 3A on CD19^+^CD25^high ^B cells**. **(**A) demonstrates the MFI of CD19^+^CD25^high^Sema3A^+ ^expression on B cells in whole blood from SLE patients compared to that of healthy controls (4.27 ± 0.47 versus 2.68 ± 0.09, *P *= 0.019). (**B**) is a representative histogram of the lower sema3A expression on B cells from SLE patients compared to that in healthy controls. The blank histogram represents the isotype control. MFI, mean fluorescence intensity; SLE, systemic lupus erythematosis.

Sema3A protein is produced by several immune cells such as B and T cells and activated monocytes. Being of a fundamental role in SLE, we considered the measurement of sema3A on B cells to be of high relevance. Importantly, the main expression of sema3A was found by us to be on CD19^+^CD25^high ^B cells (mean fluorescence intensity (MFI: 4.3), which were previously reported by us to be the B regulatory cells [23]. On the other hand, sema3A was minimally expressed on CD19^+^CD25^low ^(MFI: 1.8) *P *= 0.001. We, therefore, decided to assess the expression of sema3A and NP-1 on CD25^high ^B cells, namely, B regulatory cells. As shown in Figure 4, the level of sema3A protein was expressed lower on CD19^+^CD25^high ^B cells from SLE patients compared to CD19^+^CD25^high ^B cells from healthy controls (52.2 ± 5.8% versus 82.6 ± 6.4%, *P *< 0.0001, respectively (MFI: 2.68 ± 0.09 versus 4.27 ± 0.47, *P *= 0.019). NP-1 expression was also found to be significantly lower on CD25^high ^B cells of SLE patients when compared to that from healthy individuals (10.8 ± 3.6% versus 15.4 ± 1.4%, *P *= 0.03).

### TLR-9 expression in B cells

**Figure 5:**
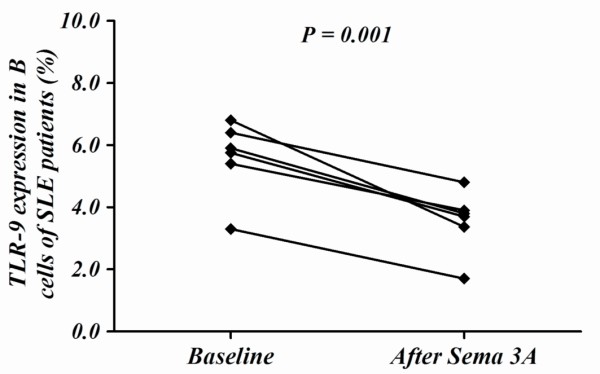
**TLR-9 expression in B cells is reduced by sema3A**. The co-culture of ODN-CpG stimulated B cells from SLE patients with sema3A reduced their TLR-9 expression by almost 50%. CpG-ODN, cytosine-phosphodiester-guanine oligodeoxynucleotides; sema3A, semaphorin 3A; SLE, systemic lupus erythematosis; TLR-9, Toll-like receptor 9.

The analysis of TLR-9 expression was performed on purified B cells from SLE patients that were incubated with or without sema3A conditioned-media. TLR-9 expression on memory B cells was significantly lower following the incubation of B cells with sema3A, by almost 50% (*P *= 0.001) compared to B cells with medium only (Figure 5).

## Discussion

The involvement of sema3A in regulating both *in vitro *and *in vivo *inflammatory responses has been previously suggested and shown to be of high relevance. The pleiotropic effects of sema3A have been shown to involve a variety of cells, such as T cells, monocytes, macrophages and endothelial cells (ECs) [24-27]. In this respect, sema3A was shown to inhibit Th1 mediated inflammatory responses in RA patients, that is, reducing the production of pro-inflammatory cytokines such as IFN-γ and IL-17 [15,20].

This study is the first in which sema3A is shown to play a role in the pathogenesis of SLE, that is, a role in humoral responses, as well as a role in modulating the autoimmune properties of B cells in SLE. Serum sema3A levels were demonstrated to be low in SLE patients, in negative correlation with disease activity, renal damage and the presence of anti-cardiolipin antibodies. It is thus suggested that sema3A is a candidate to become a useful marker for SLE disease activity and renal damage. Though of high significance, our results on sema3A serum levels in SLE patients should be strengthened with a larger number of patients in future studies. In addition, sema3A serum levels should be assessed when patients are in remission. As B cells are the source of autoantibodies and pro-inflammatory cytokine production in SLE, a correlation with sema3A expression on B cells in any relation to these activities was assessed. Indeed, it was found that sema3A expression as well as the expression of its receptor NP-1 was significantly decreased on CD19/CD25^high ^(Breg) cells from SLE patients when compared to that on this subset of cells of normal individuals.

The observation that sema3A expression is mainly present on CD19^+^CD25^high ^cells suggests sema3A as a unique marker for this subset of cells. It may be speculated that when sema3A expression is diminished, B cells may lose their regulatory signal and escape self-tolerance becoming auto-reactive and autoantibody producers. NP-1 down-regulation on B cells may also contribute to the development of B cell auto-reactivity and auto-immunity in SLE. Indeed, earlier studies have contributed to the understanding that NP-1 expression may be important for regulatory responses. In this respect, NP-1 positive CD4 T cells have been designated as CD4^+^CD25^+^Foxp3^+^NP-1^+ ^[20]. Our finding of decreased NP-1 expression, along with decreased sema3A expression on B cells in SLE further suggests that this defect may play an important role in the pathogenesis of SLE. Assuming that sema3A and NP-1 are essential for the regulatory function of B cells, one would expect to see that when their expression on B cells is decreased, B cells may shift, to become more pro-inflammatory rather that regulatory, and contribute to the development of SLE. There remains still a lack of understanding as to what causes sema3A and NP-1 to be altered in SLE. The milieu of increased pro-inflammatory cytokines such as IFN-γ and IL-6 may be a factor.

Further, sema3A has been reported to be important in the induction of the apoptosis of many immune cells, such as monocytes and macrophages, when these were found to be resistant to Fas-induced apoptosis [28]. Therefore, when sema3A expression is altered auto-reactive B cells escape apoptosis and survive to overproduce autoantibodies, thus contributing to autoimmunity in SLE. The association between TLR-9 expression in B cells, especially memory B cells and the production of IL-10 and IL-6 cytokines in SLE was previously shown by many [29-31]. The over-expression of TLR-9 in memory B cells was also shown to be in correlation with anti-dsDNA antibody production. Thus, modulation of TLR-9 expression has been suggested as a therapeutic target, aiming to reduce its expression in SLE to be followed by a reduction of IL-10 production which has been demonstrated to be associated with SLE disease activity. Considering sema3A to be an important regulator in SLE, we hypothesized that co-culturing sema3A with B cells of SLE patients could possibly reduce TLR-9 expression. Indeed, it was demonstrated in the present study that the addition of sema3A, *in vitro*, to B cells of SLE patients, significantly reduced ODN-CpG induced TLR-9 expression in memory B cells, supporting sema3A as a regulator of autoimmunity in SLE. These findings along with the observation of sema3A being reduced in SLE patients, in correlation with disease severity and autoimmunity, and memory B cells being beneficially responsive to sema3A suggest this regulatory molecule as a therapeutic agent for SLE to be assessed in the future.

## Conclusions

Sema3A is a good marker for SLE disease activity. It has a regulatory mode of action, with proven abilities of decreasing TLR9 expression in memory B cells of SLE patients. In addition, sema3A is shown to enhance regulatory properties of B cells. Further studies will focus on the therapeutic potential of sema3A in SLE.

## Abbreviations

Breg: B regulatory cells; CpG-ODN: cytosine-phosphodiester-guanine oligodeoxynucleotides; DC: dendritic cells; ECs: endothelial cells; ELISA: enzyme-linked immunosorbent assay; beta2 GPI: beta2 Glycoprotein1; HCQ: hydroxychloroquine; IL: interleukin; INFγ: interferon gamma; MFI: mean fluorescence intensity; MSCs: mesanchymal stem cells; NP-1: neuropilin-1; PBMCs: peripheral blood mononuclear cells; RA: rheumatoida arthritis; Sema3A: semaphorin 3A; SLE: systemic lupus erythematosus; SLEDAI: Systemic Lupus Erythematosus Disease Activity Index; TLR 9: Toll like receptor 9.

## Competing interests

The authors declare that they have no competing interests.

## Authors' contributions

ZV and ET participated in the design of this study, performance of all the experiments, analyzed the results and finalized the manuscript. TH assisted in the FACS analysis, designing the figures and performed the statistical analysis. KH carried out all the ELISA analyses. GS, IR, AK and DA contributed sera of their SLE and RA patients and assisted in analyzing the clinical data of these patients. OK and GN prepared in their laboratory the recombinant human semaphorin 3A condition medium and assisted in analyzing all sema3A results. All authors read and approved the final manuscript.
